# Supplementary material for: The Application of Adipose Tissue-Derived Mesenchymal Stem Cells (ADMSCs) and a Twin-Herb Formula to the Rodent Wound Healing Model: Use Alone or Together?
Source: Int J Mol Sci. 2023 Jan 10;24(2):1372. doi: 10.3390/ijms24021372 (PMC9867064; doi:10.3390/ijms24021372)
Supplement: Supplementary file 1 [file ijms-24-01372-s001.zip › ijms-2096188-supplementary.pdf]

Supplementary Table S1. List of proteins detected in the proteomic array.

Inflammation	Adiponectin, CCL2/JE/MCP-1, CCL20, CX3CL1/Fractalkine, CXCL2/GROβ, GM-CSF, ICAM-1, IFN-γ, IL-1α, IL-1β, IL-2, IL-4, IL-6, Lipocalin-2, LIX, RAGE, TNF-α, CCL4, CCL5, CCL 17, CCL 21, CCL 22, CCL 11, IL-1ra/IL-1F3, IL3, IL-13, IL17A, IL22, Lipocalin-2/NGAL, MMP-2, Neprilysin/CD10, Resistin, TIM-1/KIM-1/HAVCR.
Angiogenesis	MMP-9, VEGF, EG-VEGF, Endostatin, FGF-7, FGF acidic, HGF, TWEAK/TNFSF12
Cell proliferation and differentiation	Flt-3, Galectin-1, LIF, NOV/CCN3, WISP-1/CCN
Apoptosis	TNF-α, Clusterin, CYR61, CD26, Galectin-3, G-CSF, Osteoprotegerin/TNFRSF11B, TWEAK/TNFSF12
Others	Thymus chemokine-1, EGF, GDF, PDGF-BB, Cystatin C, Fetuin A, FGF21, Fibulin 3, Hepassocin, IGF-I, IGFBP-2, IGFBP-3, IGFBP-5, IGFBP-6, MMP-3, OPN, Pref-1/DLK1/FA1, Prolactin, RBP-4, RGMA, SCF, Serpin e1/PAI-1, VCAM-1/CD106, Ciliary neurotrophic factor, Jagged 1, MAG, G-CSF, NT-3, NT-4

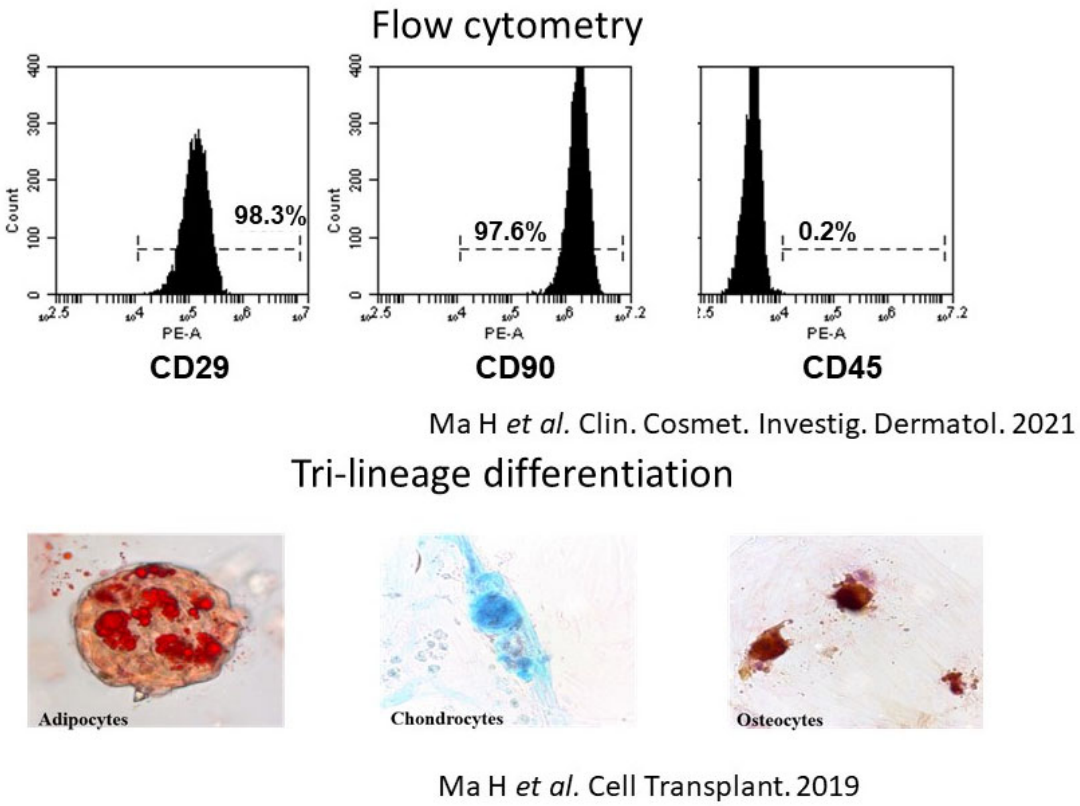

Supplementary Figure S1. Identification of MSCs by flow cytometry [9] and tri-lineage differentiation [14].
